# Activation of TAp73 and inhibition of TrxR by Verteporfin for improved cancer therapy in *TP53* mutant pancreatic tumors

**DOI:** 10.4155/fsoa-2018-0082

**Published:** 2019-01-18

**Authors:** Pilar Acedo, Aristi Fernandes, Joanna Zawacka-Pankau

**Affiliations:** 1 Department of Microbiology, Tumor & Cell Biology, Karolinska Institutet, Biomedicum, Solnavägen 9, 171 65, Stockholm, Sweden; 2 Department of Medical Biochemistry & Biophysics, Karolinska Institutet, Biomedicum, Solnavägen 9, 171 65, Stockholm, Sweden

**Keywords:** apoptosis, benzoporphyrin derivative, drug repurposing, mutant p53, pancreatic cancer, protoporphyrin IX, ROS, TAp73, thioredoxin reductase

## Abstract

**Aim:**

TAp73 is a tumor suppressor, which compensates for p53 loss and induces apoptosis in tumors in response to genotoxic stress or small-molecule treatments. Pancreatic ductal adenocarcinoma has a late onset of the disease, responds poorly to the existing therapies and has a very low survival rates.

**Result:**

Here, using drug-repurposing approach, we found that protoporphyrin IX (PpIX) and benzoporphyrin derivative (BPD) monoacid ring A activate TAp73 and induce apoptosis in pancreatic cancer cells. PpIX and BPD induce reactive oxygen species and inhibit thioredoxin reductase 1.

**Conclusion:**

Thus, PpIX and BPD target cancer cells’ vulnerabilities namely activate TAp73 tumor suppressor and inhibit oncogenic Trx1. Our findings may contribute to faster repurposing of PpIX and BPD to treat pancreatic tumors.

## Lay abstract

Despite all efforts, pancreatic cancer remains one of the most aggressive tumors. Late diagnoses, often linked with the asymptomatic disease progression, make pancreatic cancer extremely difficult to treat. We have assessed drugs that are already clinically used in the photodynamic therapy of actinic keratosis and age-related macular degeneration and showed that the compounds induce apoptotic death of cancer cells. The mechanism is via activation of the p73 tumor suppressor and inhibition of the oncogenic thioredoxin reductase. Molecules with a complementary mechanism of action inducing cell death might be very promising candidates for improved cancer therapy in pancreatic cancer patients.

## Graphical abstract

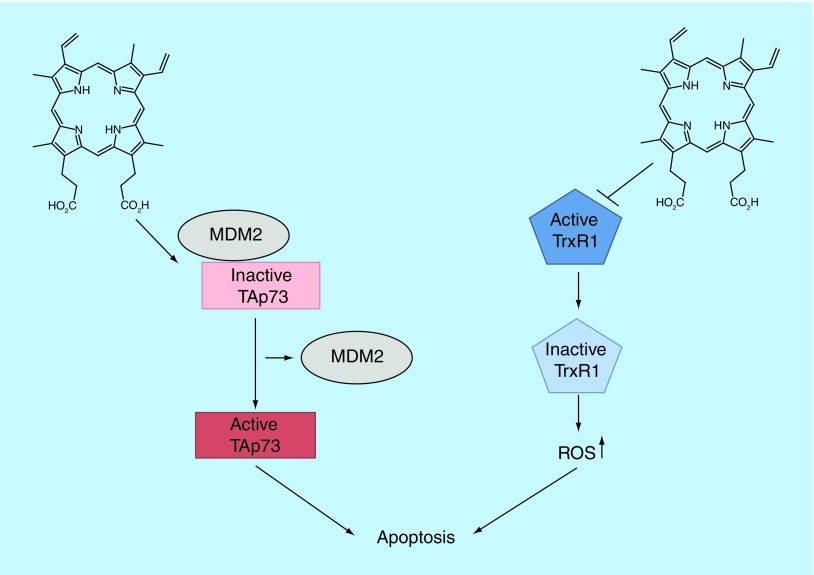

Two pathways leading to the induction of potent apoptosis by protoporphyrin IX and Verteporfin in mutant *TP53* pancreatic cancer cells. One pathway is through inhibition of TAp73/MDM2 interactions and induction of TAp73-related apoptosis and the second pathway converges on inhibition of oncogenic TrxR and accumulation of reactive oxygen species.

Pancreatic cancer is among the most lethal human cancer types and the fifth leading cause of cancer-related deaths worldwide [1]. In Europe, the death rates from pancreatic cancer continue to increase and the median survival rate for patients diagnosed with metastatic disease is only 4.6 months. Pancreatic cancer is predicted to become the second cause of cancer-related deaths in 2020 [2,3].

To date, it is still one of the most challenging solid tumors to treat. Most of the patients die within 1 year after diagnosis and only about 5% survive up to 5 years in the USA. This is mainly due to the late onset of the disease, high incidence of the metastatic stage and lack of effective treatments [4].

Treatment of pancreatic cancer is difficult and depends on the stage of the disease at the time of diagnosis. The response to currently available therapies is disappointing and the poor outcome is likely stemming from a complex set of several factors described below.

In patients not suitable for the resection with the curative intent, palliative chemotherapy may be used to improve the quality of life. Gemcitabine (GEM) was approved by the US FDA in 1998 for the treatment of patients with advanced pancreatic cancer and remains the main first-line therapy for pancreatic cancer patients, regardless of primary and acquired resistance. This resistance is partially due to DNA-repair polymorphism [5], mutations in the p53 pathway [6] or the existence of cancer stem cells [7]. Recently, combination treatment regimens with 5-fluorouracil, irinotecan and oxaliplatin (FOLFIRINOX) brought some promise in overcoming treatment resistance in patients with metastatic disease. However, pancreatic cancer remains a major unmet medical need. Thus, there is an urgent need to develop more effective treatments [8].

Pancreatic cancer is one of the cancer types in which mutant p53 impacts disease progression. *TP53* is mutated in 75% of cases of pancreatic carcinomas [9]. *TP53* gene mutations co-exist with activating mutations in classical oncogenes including *K-Ras*, which accounts for 75–90% of all pancreatic cancer cases [10].

*TP53* mutations result with the loss of p53 tumor suppressor function and preferentially promote the so-called gain of new functions. As evidenced, mutant p53 is the main driver of disease progression in pancreatic cancers. Selective therapies aiming at restoring p53 pathway to overcome the p53 loss in cancer cells, but not in normal cells, appear to be an attractive strategy for diseases such as chemotherapy-resistant pancreatic cancer. Despite the ongoing efforts, small molecules re-activating mutant p53 are still in clinical development [11].

It has been previously shown that photodynamic therapy (PDT) of cancer, which uses photoactivatable compounds and light to target cancer cells, is effective against numerous pancreatic cancer cell lines as monotherapy [12] or in combination with GEM [13] or irinotecan [14].

The majority of the clinically approved compounds used for PDT are porphyrins and their derivatives [14]. Protoporphyrin IX (PpIX) is a metabolite of γ-aminolevulinic acid, a pro-drug applied in PDT of cancer [14]. PpIX itself, without light excitation, was shown to induce wild-type (wt) p53-related cell death in several human cancer cell lines including human colon carcinoma [15]. Interestingly, it has recently been demonstrated that PpIX stabilizes and activates TAp73 and induces TAp73-dependent apoptosis in cancer cells lacking *TP53* [16].

TrxR1 is a selenoprotein of the thioredoxin system and is central in maintaining redox homeostasis in cells [17]. TrxR has a protective role against oxidative stress and contributes to tumor progression once the disease has already developed. Thus, *TXNRD1* is often overexpressed in cancers and is a promising target for improved cancer therapy [17].

Here, we found that PpIX and benzoporphyrin derivative (BPD; verteporfin) induce TAp73 and its pro-apoptotic targets on mRNA and protein levels. Next, it was demonstrated that PpIX and BPD induce reactive oxygen species (ROS) and antioxidant response genes *HMOX-1* and *NQO1*. PpIX and BPD inhibited TrxR1 *in vitro* and in pancreatic cancer cells harboring mutant p53. Taken together, our data add up to the mechanism by which PpIX and now BPD induce apoptosis in cancer cells harboring *TP53* gene mutations, which is via reactivation of TAp73 tumor suppressor and inhibition of TrxR1.

## Materials & methods

### Cell culture

Human pancreatic cancer cell lines, Paca3 (wt *TP53*), MiaPaca2 (mutant *TP53R248W*) and Panc1 (mutant *TP53R273H*) were kindly provided by Dr Rainer Heuchel from Karolinska Institutet, Sweden. Mouse pancreatic ductal cancer cell line Panc02 (wt *TP53*) was provided by Dr Maximilian Schnurr from University of Munich, Germany and made available by Dr Yihai Cao from Karolinska Institutet, Sweden.

Normal human pancreatic ductal epithelial cells (HPDE) immortalized by HPV transformation [18] were also provided by Dr Heuchel. Cells were maintained in DMEM medium supplemented with 10% fetal bovine serum and 1% penicillin/streptomycin solution (Sigma-Aldrich, Munich, Germany). The cells were cultured at 37°C in a humidified incubator with 5% CO_2_. The cells were routinely checked for mycoplasma during the whole duration of the project.

### Chemicals

PpIX and BPD were purchased from Sigma-Aldrich dissolved in 100% DMSO (Sigma-Aldrich) to a final concentration of 2 mg/ml and stored in dark in amber tubes at room temperature (PpIX) or at -20°C (BPD) until further use.

Cisplatin (cis-diamminedichloridoplatinum(II), CDDP; Sigma-Aldrich) was dissolved in 0.9% NaCl to a concentration of 25 mM and used at final concentration of 20 μM. GEM (2′,2′-difluoro-2′-deoxycytidine, GEM; Abcam, Cambridge, UK) was prepared as 1 mM stock solution in 0.9% NaCl. Resveratrol (Enzo Biochem, NY, USA) was reconstituted to 40 mM in 100% DMSO, aliquoted and stored at −20°C.

MTT reagent (Sigma-Aldrich) was reconstituted in PBS (prepared from 1× tablets Sigma-Aldrich) to 5 mg/ml, filter-sterilized and stored at +4°C for a week or at -20°C until further use.

### Verteporfin uptake & cellular localization

Cells were incubated with 2.5 μg/ml of PpIX or BPD reconstituted in cell culture media for 3 h, washed three times with PBS and co-stained with ER-Tracker™ Green for live-cell imaging (Molecular Probes, OR, USA) according to manufacturer protocol. 4′,6-diamidino-2-phenylindole (DAPI; Molecular Probes) was used for nuclear counterstaining. An organelle marker was excited using a 488 nm laser and BPD was excited with 420 nm (emission at 690 nm). Images were taken using an Olympus IX-71 microscope and DeltaVision SoftWoRx.

### Cell viability

Cell viability was assessed by MTT assay as provided by the manufacturer (Sigma-Aldrich) and as described previously [15]. Briefly, 3000 cells were seeded in 96-well plate and allowed to adhere overnight. The cells were treated with the increasing concentrations of the compounds (PpIX or BPD) or 25 or 5 μM cisplatin or GEM for 72 h and the viability was measured using MTT reagent according to the manufacturer protocol.

### Real-time PCR

Quantitative PCR was performed as described previously [19]. Cells were seeded in 6-well plates, allowed to adhere for 24 h and treated with 2.5 μg/ml PpIX or BPD for 8 h. After isolation, mRNA was reverse transcribed to cDNA according to the manufacturer's instructions (Bio-Rad, Solna, Sweden). For qPCR reaction 150 nM, 10 ng cDNA, 7.5 μl 2 × master mix (Bio-Rad) and water to a total of 15 μl were used. Primers used: *GAPDH* forward: TCATTTCCTGGTATGACAACG and reverse: ATGTGGGCCATGAGGT, *NOXA* forward 5′-AAGTGCAAGTAGCTGGAAG-3′, reverse: 5′-TGTCTCCAAATCTCCTGAGT-3′, *PUMA* forward: 5′-CTCAACGCACAGTACGAG-3′ and reverse: 5′-GTCCCATGAGATTGTACAG-3′, *HMOX-1* forward: 5′-TTCACCTTCCCCAACATTGC-3′ and reverse: 5′-TATCACCCTCTGCCTGACTG-3′, *Bid* forward: 5′-GTGAGGTCAACAACGGTTCC-3′ and reverse: 5′-TGCCTCTATTCTTCCCAAGC-3′, *Bim* forward: 5′-TGGCAAAGCAACCTTCTGATG-3′ and reverse: 5′-GCAGGCTGCAATTGTCTACCT-3′, *Bax* forward: 5′ – GCTGTTGGGCTGGATCCAAG – 3′ and reverse: 5′ – TCAGCCCATCTTCTTCCAGA – 3′.

### Thioredoxin reductase activity assay

For the enzymatic activity of purified TrxR1 we followed the protocol previously described [20,21]. Briefly, 10 nM of purified TrxR1 [22] was pre-incubated with BPD at the indicated concentrations (0–20 μM) in 50 μl phosphate EDTA (PE) buffer supplemented with 150 μM NADPH for 30 min at room temperature. Next, 2.5 mM DTNB and 150 μM NADPH were added to the samples just before measurement. DTNB reduction was performed for 6 min by detection of TNB anion formation detected as a change in the absorbance at 412 nm.

For cellular TrxR activity, Panc1 and MiaPaCa2 cells were treated with 2.5 μg/ml PpIX or BPD for 6 h, after which they were harvested and resuspended in a Tris-EDTA (TE) buffer containing a protease inhibitor cocktail (complete, mini protease cocktail tablets, Roche, Solna, Sweden). Cells were sonicated and the total protein concentration of the supernatant was determined using a Bradford reagent kit (Bio-Rad Laboratories, Solna, Sweden). Cellular TrxR1 activity was determined using the previously described endpoint Trx-dependent insulin reduction assay [20]. Briefly, total cellular protein (20 μg) was incubated with 15 μM recombinant human wt Trx in the presence of 297 μM insulin, 1.3 mM NADPH, 85 mM Hepes buffer (pH 7.6) and 13 mM EDTA for 30 min at 37°C, in a total volume of 50 μl. The reaction was stopped by the addition of 200 μl of 7.2 M guanidine–HCl in 0.2 M Tris–HCl (pH 8.0) containing 1 mM DTNB. The activity was then determined by measuring absorbance at 412 nm using a VersaMax microplate reader (Molecular Devices, CA, USA) with a background absorbance reference for each sample, containing all components except purified TrxR, incubated and treated in the same manner.

### Cell death detection

Propidum iodide (PI) and FITC-Annexin V (from BD Biosciences, CA, USA) staining was performed according to the manufacturer's protocols and FACS analysis was carried out using the CELLQuest software (CELLQuest, NJ, USA) as described previously [23].

### ROS measurement

Cells were treaded with compounds for 16 h, washed with PBS and then the generation of ROS assessed by flow cytometry. ROS were detected using 2′,7′-dichlorofluorescein (H_2_DCF; Sigma-Aldrich) and method described previously [21].

Hydroethidine staining was performed according to the manufacturer protocol (Life Technologies, Stockholm, Sweden). Data analysis was performed with the CELLQuest software (CELLQuest, BD Biosciences, Franklin Lakes, NJ, USA).

### Western blotting

Western blotting was performed according to the standard protocol. Briefly, 100 μg of total cell lysate was subjected to electrophoresis, after transfer the following antibodies were used to detect proteins: anti-TAp73 (A300-126A) (Bethyl Laboratories, TX, USA), anti-BAX (N-20; Santa Cruz Biotechnology, Germany) anti-PUMA (ABC158; Merck, MA, USA), anti-HO-1 (H-105; Santa Cruz Biotechnology), anti-NRF2 (H-300; Santa Cruz Biotechnology), anti-Bid (FL-195; Santa Cruz Biotechnology, TX, USA), anti-PARP (F-2; Santa Cruz Biotechnology), anti-β-actin (A2228; Sigma-Aldrich).

## Results

### BPD localize into the cytoplasm of Panc1 cancer cells

Pancreatic ductal adenocarcinomas (PDACs) are cancers of poor clinical outcome due to the development of chemoresistance and disease relapse with mutant p53 being associated with the aggressiveness of the disease. Whether the re-activation of TAp73 can compensate for the p53 loss in cancers with the *TP53* gain-of-function mutations has not been unequivocally demonstrated yet. Therefore, we tested the outcome of TAp73 activation in PDAC cancer cell lines harboring hot-spot *TP53R273H* mutation.

**Figure 1.**
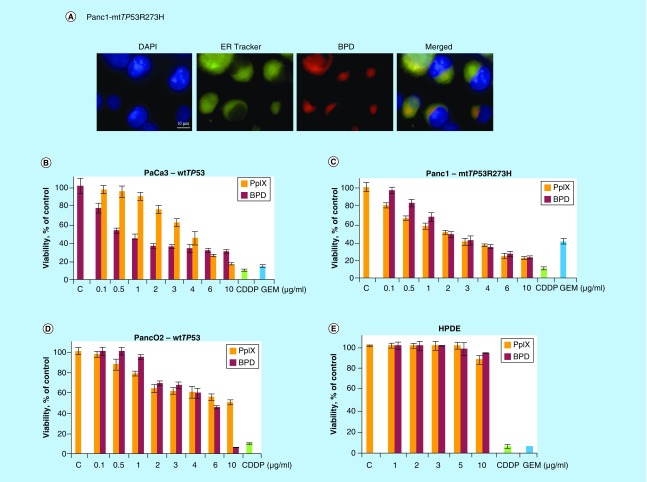
**Benzoporphyrin derivative accumulates in cancer cells and inhibits proliferation in a dose-dependent manner.** **(A)** Fluorescence microscopy shows that benzoporphyrin derivative (BPD) accumulates largely in the cytoplasm and partially in endoplasmic reticulum in Panc1 pancreatic cancer cells. Nuclei were stained with DAPI and mitochondria and endoplasmic reticulum with ER-Tracker Green, respectively. The image is a representative data of three independent experiments. **(B)** PpIX (orange bars) and BPD (red bars) inhibit proliferation of PaCa3 (wt*TP53*), Panc1 (mt*TP*53*R273H*) **(C)** and Panc02 **(D)** pancreatic cancer cells as assessed by MTT assay. Cells were treated for 72 h, 25 μM cisplatin (CDDP, green bars) and 5 μM gemcitabine (GEM, blue bars) were used as positive controls of growth inhibition. **(E)** PpIX (orange bars) and BPD (red bars) do not inhibit proliferation of normal, nontransformed ductal epithelial (HPDE) cells as assessed by MTT assay. PpIX: Protoporphyrin IX.

First, we assessed the subcellular localization of BPD in Panc1 mutant *TP53R273H*-expressing cancer cells (Figure 1A). Fluorescence microscopy indicated that BPD (red fluorescence emission) is uptaken to high degree by cancer cells 3 h after incubation with the drug. Next, we found that verteporfin localized in large in cytoplasm and partially in the endoplasmic reticulum. This is in agreement with the previously published data.

### PpIX & BPD inhibit proliferation of pancreatic cancer cells but not of nontransformed human ductal epithelial cells

To assess the impact of PpIX and BPD on the proliferation of pancreatic cancer cells we performed MTT viability assay. The standard treatment for the advanced pancreatic cancer includes monotherapy with GEM. Therefore, we used GEM in the most of the experiments for the comparative purposes.

MTT showed that PpIX and BPD inhibit cancer cells’ proliferation in a dose-dependent fashion (Figure 1B–E). In agreement with the previous data, cancer cells expressing wt p53 protein (PaCa3) were sensitive to 72 h-treatment with PpIX [15] (Figure 1B) and showed even higher growth inhibition rate after BPD treatment. Interestingly, mutant p53-harboring cells (Panc1) were more sensitive to PpIX than wtp53-expressing PaCa3 cells and slightly less sensitive to BPD (Figure 1C).

The calculated IC_50_ value for PpIX was 4.4 μM for PaCa3 (wt*TP53*) cells and 3.5 μM for Panc1 (mt*TP53R273H*) and 2.2 and 2.8 μM for BPD, respectively.

Of note, murine (Panc02) and human (MiaPaca2) pancreatic cancer cells were less sensitive to PpIX and BPD at the concentrations tested (Figure 1D and data not shown) and normal human epithelial ductal cells were insensitive (Figure 1E). On the other hand, all cell lines applied in the study were highly susceptible to low dose (25 μM) cisplatin and 5 μM GEM.

Consistent with the previous data, wtp53 cancer cells were sensitive to PpIX [15]. Interestingly, mutant p53 Panc1 cancer cells were the most sensitive among the cell lines tested. Based on the above and previous studies, we selected 2.5 μg/ml concentration for further studies on the cell death induction upon reactivation of TAp73 in mutant p53 pancreatic cancer cells.

### PpIX & BPD induce apoptosis in PDAC cells

**Figure 2.**
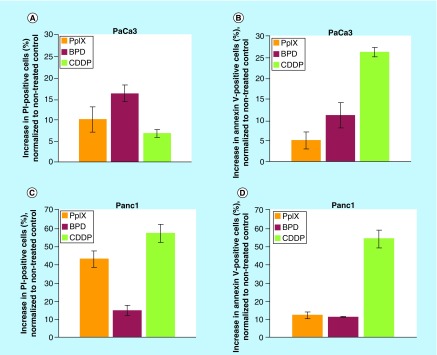
**Protoporphyrin IX and benzoporphyrin derivative activate early and late apoptosis in pancreatic cancer cells.** **(A)** Treatment with 2.5 μg/ml PpIX (orange bars) and BPD (red bars) for 48 h induced late (PI staining) and early apoptosis (FITC-Annexin V) **(B)** in wtp53-harboring PaCa3 pancreatic cancer cells (n = 3). 20 μM CDDP (green bars) was used as a positive control for apoptosis induction. **(C)** Treatment with 2.5 μg/ml PpIX and BPD for 48 h induced late (PI staining) and early apoptosis (FITC-Annexin V) **(D)** in mt*TP53*-harbouring Panc1 pancreatic cancer cells (n = 3). 20 μM CDDP was used as a positive control for apoptosis induction. BPD: Benzoporphyrin derivative; PI: Propidum iodide; PpIX: Protoporphyrin IX.

To investigate if the growth inhibition observed in the MTT viability assays was a consequence of the induction of apoptosis, we treated PaCa3 and Panc1 cells for 48 h with 2.5 μg/ml PpIX or BPD and performed flow cytometry analysis using PI and Annexin V stainings. We found that Panc1 undergoes high late apoptosis after PpIX treatment, as demonstrated by above 40% increase of PI positive cells relative to the untreated control. At this time-point, Annexin V positivity, indicating early apoptosis, increased by 12 or 9% after PpIX or BPD treatments, respectively (Figure 2C & D). In the PaCa3 cells harboring wt*TP53*, we observed 10 and 17% increase in PI positive cells after PpIX or BPD treatment, respectively. The increase in Annexin V-positive cells was 5% for PpIX and 11% for BPD. Thus, both compounds induce early and late apoptosis in the tested cancer cell lines. Cisplatin, used as a positive control, induced high early apoptosis in PaCa3 (wt*TP53*) cells and early and late apoptosis in Panc1 (mt*TP53*) cells.

### PpIX & BPD induce TAp73 & its pro-apoptotic targets

**Figure 3.**
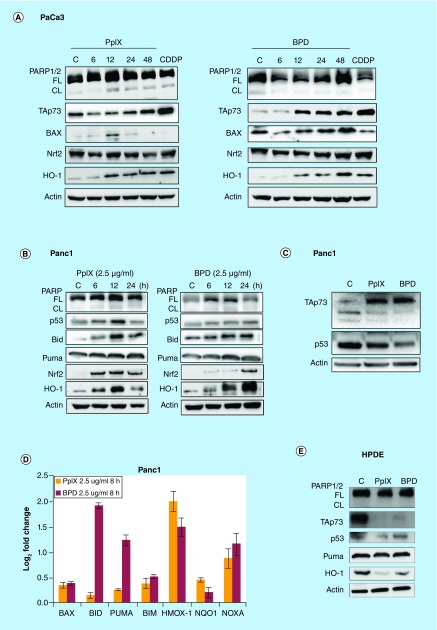
**Reactivation of TAp73 by protoporphyrin IX and benzoporphyrin derivative induces apoptosis in pancreatic cancer cells.** **(A)** Protoporphyrin IX (PpIX) (2.5 μg/ml) induce PARP cleavage in PaCa3 and Panc1 cells and BPD in Panc1 pancreatic cancer cells **(B & C)**. The effect is time dependent. The accumulation of cleaved PARP correlates with the stabilization of TAp73 tumor suppressor protein levels and its pro-apoptotic target Bax. Both compounds upregulated HO-1 and its transcriptional regulator Nrf2. 20 μM CDDP was used as a positive control for apoptosis induction. **(D)** Treatment with PpIX (orange bars) and BPD (red bars) increased the mRNA levels of apoptotic TAp73 targets and the antioxidant response proteins in Panc1 pancreatic cancer cells. **(E)** PpIX and BPD treatment did not induce TAp73 or its proapoptotic targets in non-transformed HPDE. BPD: Benzoporporphyrin derivative; CL: Cleaved; FL: Full length; HPDE: Human pancreatic ductal epithelial cells; HO-1: Heme oxygenase.

It has been demonstrated that PpIX reactivates TAp73 tumor suppressor and induces TAp73-dependent apoptosis in *TP53*-null cancer cells [16]. To assess if the growth inhibition observed in pancreatic cancer cells upon treatment with PpIX and BPD is a result of the ongoing apoptosis, we evaluated the expression of TAp73 tumor suppressor and its pro-apoptotic targets in PaCa3 and Panc1 cancer cells. Western blotting showed that in the dark, both PpIX and BPD induce the levels of endogenous TAp73, which correlates with the accumulation of its proapoptotic targets PUMA, Bax and Bid (Figure 3A–C) in both cell lines. Time-dependent accumulation of the cleaved PARP was detected in Panc1 cells. Next, in Panc1 cells, PpIX and BPD upregulated the expression of *bax*, *bid*, *puma* and *noxa* genes (Figure 3D). This indicates that apoptosis is due to the activation of TAp73 tumor suppressor (Figure 3A & C). Interestingly, we detected upregulation of the antioxidant response proteins, HO-1 and Nrf2 (Figure 3A–D). PpIX and BPD did not induce PARP cleavage or accumulation of TAp73 and its proapoptotic targets in nontransformed HPDE cells at the concentrations tested (Figure 3E). We have observed downregulation of TAp73 after PpIX and BPD treatment; however, the mechanism remains to be elucidated.

### PpIX & BPD induce ROS in PDAC

**Figure 4.**
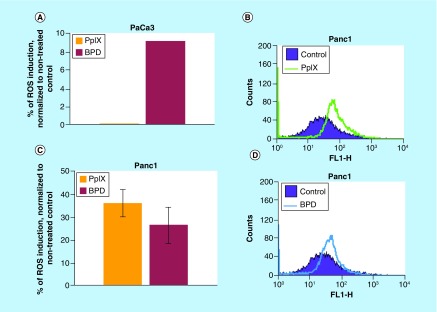
**Protoporphyrin IX and benzoporphyrin derivative induce ROS in pancreatic cancer cells.** **(A)** BPD treatment upregulates ROS levels in PaCa3 cells as estimated by the 2',7′-dichlorodihydrofluorescein diacetate staining after 16 h post-treatment (n = 3). **(B–D)** Both PpIX and BPD induced ROS in Panc1 cells after 16 h, as assessed by the 2',7′-dichlorodihydrofluorescein diacetate staining (n = 3). BPD: Benzoporphyrin derivative; PpIX: Protoporphyrin IX.

Since we detected accumulation of the antioxidant response proteins after treatment with PpIX and BPD in pancreatic cancer cells; we thus sought to investigate if PpIX and BPD elevate ROS. We assessed the levels of ROS using DCFDA and HE probes. Figures 4A–C show that PpIX and BPD induced ROS levels in PaCa3 and Panc1 cells. Pretreatment with the ROS scavenger resveratrol inhibited the generation of ROS induced by PpIX and BPD in Panc1 cells (Supplementary Figure 1A). Importantly, the compounds did not induce ROS in HPDE cells (Supplementary Figure 1B).

### PpIX & BPD are inhibitors of thioredoxin reductase

PpIX was first identified as an inhibitor of thioredoxin reductase in an *in vitro* screen for the inhibitors of the enzymes [24]. Since we observed significant induction of ROS in cancer cells treated with PpIX or BPD and inhibition of TrxR is known to cause accumulation of ROS, we reasoned that apart from TAp73-dependent apoptosis, PpIX and BPD inhibit TrxR which might explain the elevated ROS levels detected in the treated cancer cells.

**Figure 5.**
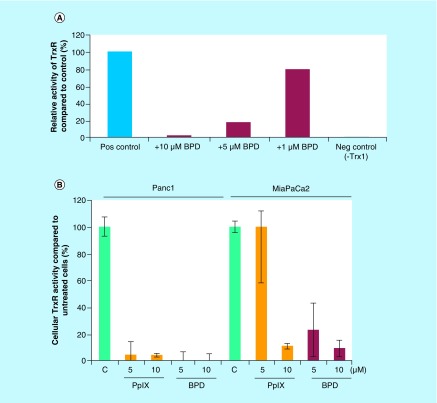
**Protoporphyrin IX and benzoporphyrin derivative inhibit thioredoxin reductase activity.** **(A)** BPD (red bars) inhibits the activity of the recombinant TrxR. The representative data of three independent experiments is presented. **(B)** Both PpIX (orange bars) and BPD (red bars) inhibit TrxR in Panc1 and MiaPaCa2 pancreatic cancer cells (n = 2). BPD: Benzoporphyrin derivative; PpIX: Protoporphyrin IX.

As demonstrated in Figure 5A, recombinant TrxR was inhibited by both, PpIX and BPD. To assess if PpIX and BPD inhibit TrxR activity in cancer cells also, we treated Panc1 cells with the increasing concentrations of PpIX or BPD for 6 h and assayed the activity using the end point Trx-dependent insulin-reduction assay. PpIX and BPD treatment inhibited TrxR1 in Panc1 cancer cells (Figure 5B) as well as in other mutant p53 harboring cell line, MiaPaCa2 (mt*TP53R248W*). Taken together, these data demonstrate that BPD, similarly to PpIX, is a potent inhibitor of TrxR in pancreatic cancer cells.

## Discussion

Several tyrosine kinases inhibitors were reported to have off-target effects. For example, BRAF inhibitor, vemurafenib, used for treating metastatic melanoma [25] with overall good clinical outcome [26], was manifested to induce unusual photosensitivity in patients [27]. A recent study by Klaeger *et al*. [28] showed that several kinase inhibitors, including vemurafenib, in addition to their designed targets effectively inhibit ferrochelatase, the last enzyme in the heme biosynthesis. The authors speculated that the accumulation of PpIX, resulting from inhibition of ferrochelatase might lead to the photosensitivity of the patients upon treatment with kinase inhibitors.

PpIX is a metabolite of γ-aminolevulinic acid, heme precursor and a pro-drug applied in the PDT of cancer [14]. We have shown in a drug-repurposing approach that PpIX re-activates wt p53 and TAp73 tumor suppressors and induces p53- or TAp73-dependent apoptosis in cancer cells. The mechanism of activation of p53 and TAp73 is via inhibition of p53/MDM2 and TAp73/MDM2(X) interactions [15,16]. Next, PpIX was identified in the *in vitro* screen as a potent, competitive inhibitor of thioredoxin reductase (TrxR) [24]. Thus, here we speculated that PpIX, exogenously delivered to pancreatic cancer cells harboring *TP*53 mutations, will induce apoptosis via activation of the p53 protein family member, TAp73. We also reasoned that PpIX and its clinically applied analog BPD may simultaneously inhibit TrxR in pancreatic cancer cells.

The detailed analysis revealed that PpIX and BPD, known under the commercial name of Verteporfin, inhibit proliferation of pancreatic cancer cells without affecting nontransformed cells. The mechanism of growth inhibition was by the induction of apoptosis. Next, both compounds triggered antioxidant response and induced accumulation of ROS only in cancer cells but not in normal HPDE cells. The above prompted us to investigate if the robust induction of ROS is a consequence of the inhibition of TrxR. According to our findings, BPD inhibited the enzymatic activity of the recombinant thioredoxin reductase. Both PpIX and BPD inhibited the enzyme in Panc1 and MiaPaCa2 cancer cells as demonstrated by the Trx-dependent endpoint insulin reduction assay.

Inhibitors of TrxR are currently under preclinical development and are promising candidates for improved cancer therapy [29]. Several small molecules activating wt or mutant p53 were also shown to inhibit TrxR [21,30,31]. The small molecule APR-246, a mutant p53-reactivating compound in Phase II clinical development, is a pro-drug, converted in cells to methylene quinuclidinone [32]. Methylene quinuclidinone is a Michael acceptor and was demonstrated to target cysteines in the p53 core domain [33] and selenocysteine (Sec) residues in the C-terminal motif of TrxR1 [30]. Our findings demonstrate that PpIX and BPD inhibit TrxR. The mechanism by which this occurs remains to be elucidated. Structural studies on the heme-binding protein, heme oxygenase 2 (HO-2), revealed that in the oxidized state of cysteine residues in apoprotein, heme binds 2.5-fold more tightly than in the reduced state [34]. This, together with the finding that the Sec-to-Cys mutant of TrxR1 was resistant to inhibition by PpIX [24], strongly implies that Sec residue in TrxR might be the binding site of PpIX and BPD (Verteporfin).

We showed that PpIX and now also BPD activates TAp73 in pancreatic cancer cells, which results in the induction of apoptotic cell death. In parallel, the compounds induce ROS, which contributes to cell killing. Induction of ROS levels is with high probability due to the inhibition of TrxR by PpIX and BPD.

## Future perspective

Our findings might have an important clinical implication and could support fast repurposing of porphyrins in treating pancreatic cancer patients harboring *TP*53 mutations. Dual targeting of TAp73 and TrxR by small molecules that we have identified might bring therapeutic advantage in overcoming the development of the treatment-resistant disease. In particular, we foresee that the parallel activation of TAp73 and inhibition of TrxR will significantly increase the stress burden in already stressed cancer cells when compared with separate approaches, which will contribute to the selective tumor killing without affecting normal cells.

**Summary points**

- Protoporphyrin IX (PpIX) and benzoporphyrin derivative (BPD) induce apoptosis in pancreatic ductal adenocarcinoma cells by activating TAp73.
- PpIX and BPD generate reactive oxygen species in pancreatic cancer cells.
- PpIX and BPD inhibit thioredoxin reductase *in vitro* and in cancer cells.
