# Supplementary material for: Activation of TAp73 and inhibition of TrxR by Verteporfin for improved cancer therapy in TP53 mutant pancreatic tumors
Source: Future Sci OA. 2019 Jan 18;5(2):FSO366. doi: 10.4155/fsoa-2018-0082 (PMC6391631; doi:10.4155/fsoa-2018-0082)
Supplement: Supplementary file 1 [file fsoa-05-366-s1.docx]

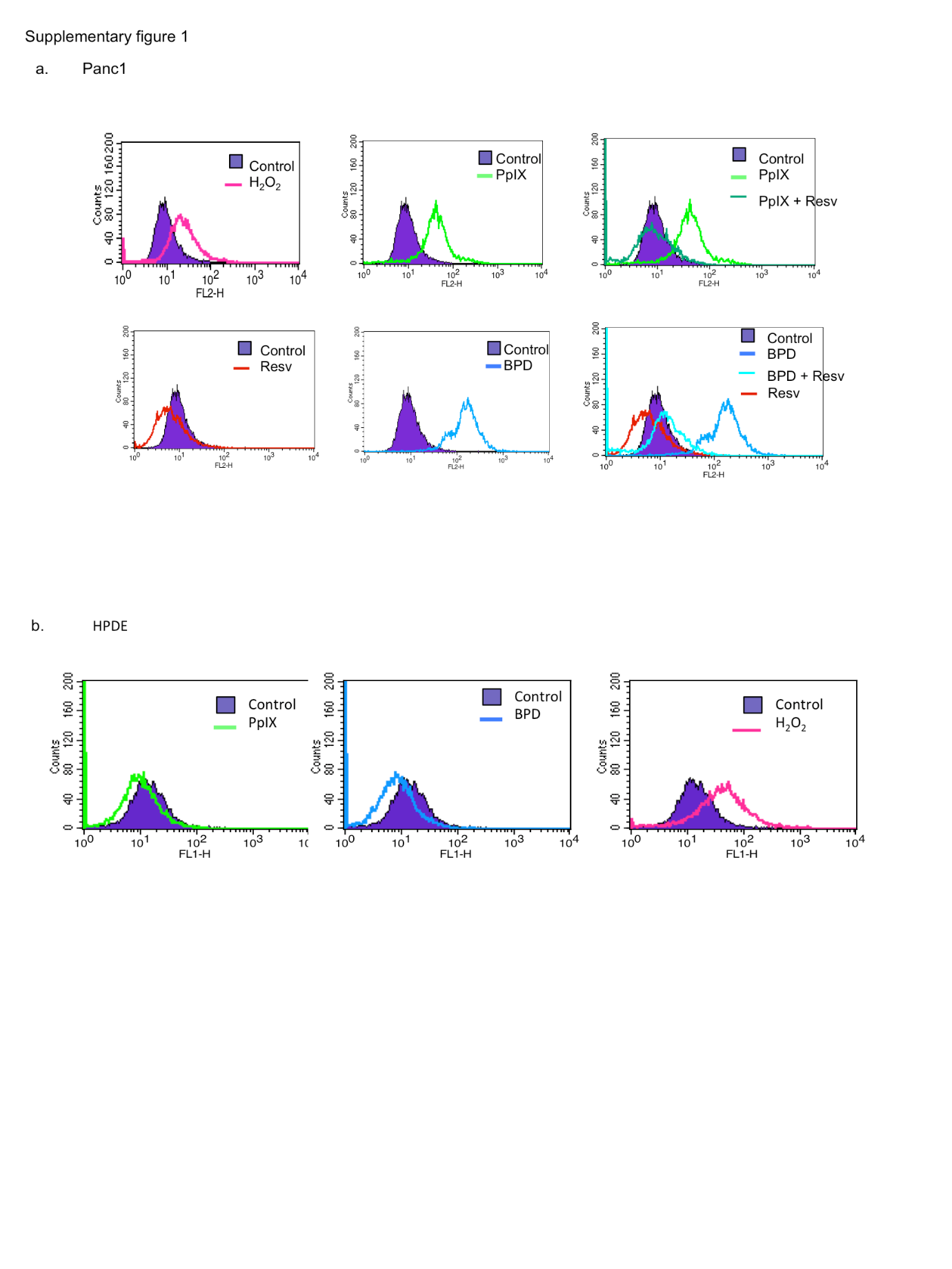


**Supplementary Figure 1. PpIX and BPD induce ROS generation.**

1. PpIX and BPD generated ROS in Panc1 cells as assessed by HE staining. Pre-treatment with 4 μM resveratrol reverted the effect of these compounds. Representative histograms of three independent experiments are shown.
2. Neither, PpIX nor BPD induced ROS in non-transformed HPDE cells (DCFDA staining). H_2_O_2_ treatment was used as positive control of ROS induction. The representative histograms are shown (n=3).
